# Entomological Assessment of *Onchocerca* Species Transmission by Black Flies in Selected Communities in the West Region of Cameroon

**DOI:** 10.3390/pathogens9090722

**Published:** 2020-09-02

**Authors:** Cabirou Mounchili Shintouo, Joel Ebai Nguve, Fru Bertha Asa, Robert Adamu Shey, Joseph Kamga, Jacob Souopgui, Stephen Mbigha Ghogomu, Rose Njemini

**Affiliations:** 1 Department of Gerontology, Faculty of Medicine and Pharmacy, Vrije Universiteit Brussel, Laarbeeklaan 103, B-1090 Brussels, Belgium; Cabirou.Mounchili.Shintouo@vub.be; 2 Frailty in Ageing Research Group, Vrije Universiteit Brussel, Laarbeeklaan 103, B-1090 Brussels, Belgium; 3 Department of Biochemistry and Molecular Biology, Faculty of Science, University of Buea, Buea P.O. Box 63, Cameroon; joelebai@gmail.com (J.E.N.); robeshey@ulb.ac.be (R.A.S.); josephkamga69@yahoo.fr (J.K.); 4 Department of Public Health and Hygiene, Faculty of Health Science, University of Buea, Buea P.O. Box 63, Cameroon; bertizi.ba@gmail.com; 5 Department of Molecular Biology, Institute of Biology and Molecular Medicine, IBMM, Universite Libre de Bruxelles, Gosselies Campus, 126040 Gosselies, Belgium

**Keywords:** onchocerciasis, ivermectin, O-150 PCR, poolscreen, elimination

## Abstract

The enormity of the public health burden of onchocerciasis motivated the creation of various large-scale control programs that have depended principally on mass treatment of endemic communities with ivermectin for the elimination of the disease. Parasitological evaluation of *Onchocerca* species in the West Region of Cameroon indicates significant progress in the interruption of parasite transmission in some communities under ivermectin treatment. However, to verify the complete elimination of onchocerciasis, entomological assessment through O-150 PCR poolscreen of black flies is mandatory. Thus, in the present study, we assessed transmission of *Onchocerca* species using an O-150 PCR technique to screen pools of black flies—in seven onchocerciasis endemic communities (Makouopsap, Bankambe, Lemgo, Tsesse, Ndionzou, Kouffen, and Bayon) in Cameroon. Two thousand black flies were assessed—in each community—for the presence of *Onchocerca* species DNA. Our results show that the frequency of infective flies was 0.6% in Makouopsap and 0.0% in the other communities. On the other hand, the frequency of infected flies was 0.8% in Makouopsap, 0.2% in Bankambe, 0.1% in Bayon, and 0.0% in Lemgo, Tsesse, Ndionzou, and Kouffen. These results provide entomologic evidence for continuous transmission of *Onchocerca* species in Makouopsap, risk of active transmission in Bankambe, and Bayon, and a suppressed transmission in the four other studied communities.

## 1. Introduction

Human onchocerciasis, also known as river blindness, is a filarial disease caused by the parasite *Onchocerca volvulus* (*O. volvulus*) and is one of the most debilitating yet neglected tropical diseases [1]. The infective larvae (L3) are transmitted through the repeated bites of infective black flies (*Simulium*), which eventually give rise to adult worms. The latter dwell in subcutaneous tissues in humans where they can survive for about 15 years under drug pressure, with adult females hatching about 1600 microfilariae daily [2]. The microfilariae migrate through the skin where they can be ingested by a black fly during a blood meal. Once ingested, the microfilaria migrates to the thoracic muscles of the black fly where it develops into the first (L1) and second (L2) larval stages, and the black fly becomes infected. The L2 larva subsequently develops into the L3 stage, which migrates to the black fly’s proboscis rendering it infective and, as such, the fly can infect humans when taking a blood meal [3]. On the other hand, *Onchocerca ochengi* (*O. ochengi*), a bovine parasite of Zebu cattle in Africa is the closest related species to *O. volvulus* and both parasites are transmitted by the same black fly vector [4]. Whereas *O. ochengi* causes little harm in its cattle host, the microfilariae of *O. volvulus* induce considerable pathology in humans [5]. The *O. volvulus* microfilariae cause persistent itching, severe skin diseases, and frequently induce ocular lesions that may result in a progressive loss of vision and blindness [6]. In 2017, about 20.9 million persons were infected with human onchocerciasis worldwide, with 1.15 million having vision loss, 14.6 million having a skin disease, and more than 99% living in Africa [7]. In Cameroon where this study was conducted, 2.8 million people are infected by the disease and 5.2 million people are at risk of infection [8], making the disease an important public health concern.

To mitigate the public health and socio-economic burden of onchocerciasis, various large-scale control programs—through Community Directed Treatment with Ivermectin (CDTI)—have been implemented in onchocerciasis endemic regions in Africa [9]. In the West Region of Cameroon, treatment started in 1996—with the support of the Carter Center and Lions Club International Foundation—as a community-based treatment with an annual dose of ivermectin [10]. This CDTI program in the West Region of Cameroon was evaluated by the World Health Organization (WHO) and the African Program for Onchocerciasis Control (APOC) in 2011, and the results of the survey revealed a microfilaria prevalence of 38.6% [11]. Therefore, the conduction of additional surveys at intervals of three to four years was highly recommended. In this framework, Kamga et al. [12], repeated the survey in 2015 and concluded that, with the exception of Makouopsap, there was an important progress in parasite transmission interruption in the communities surveyed. However, to guarantee complete elimination of onchocerciasis, poolscreen testing of black flies is strongly recommended by WHO [13].

In the current study, we aimed to further evaluate the progress made towards eliminating onchocerciasis transmission in the West Region of Cameroon, by focusing on the entomological assessment of *Onchocerca* species (*Onchocerca* spp.) transmission by black flies using a O-150 PCR (Poolscreen) technique in selected communities under ivermectin treatment for the past two decades.

## 2. Results

### 2.1. Percentage of Infective and Infected Pools of Black Flies in Surveyed Communities

**Figure 1:**
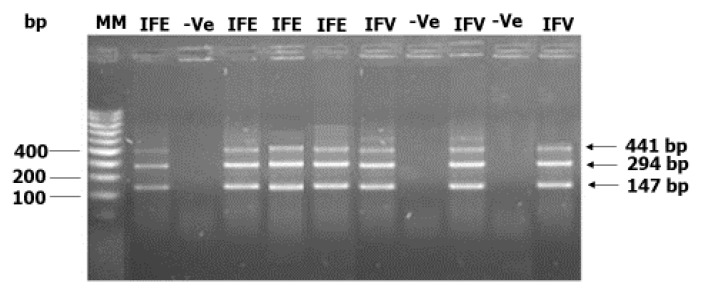
PCR analysis of infective and infected black fly pools in Makouopsap. The PCR products were amplified using specific sets of primers. The amplified PCR products were separated on 2% agarose gels and visualized under UV light. MM = Molecular weight maker, IFE = Infected pool, IFV = Infective pool, −Ve = Negative pool, and bp = base pair.

**Figure 2:**
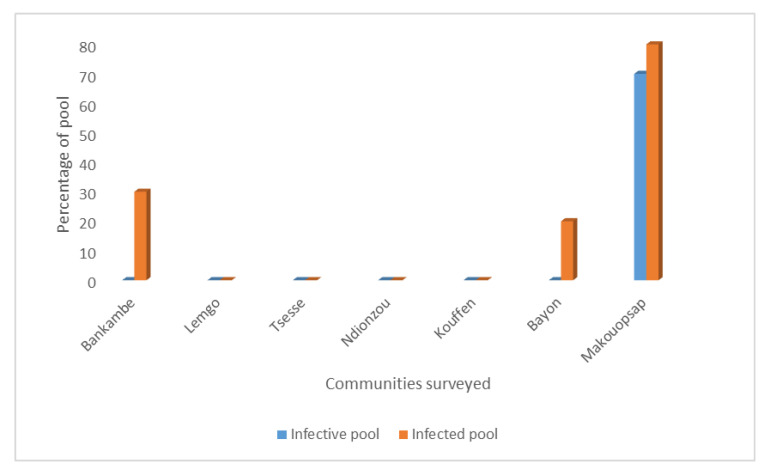
Summary of infective and infected pools of black flies in the surveyed communities. The percentage of the infective and infected pools of black flies were gotten from the agarose gel electropherogram of an O-150 PCR assay. Overall, 7 (10%) and 13 (18.6%) of the pools were infective and infected, respectively. Although there was a significant variation in infective status of black flies across the communities (*p* < 0.001), the community of sampling was not a significant risk factor (*p* = 0.992) for the infective status of black flies overall following logistic regression analysis. However, the community of sampling was a significant risk factor for infected status of black flies (*p* = 0.007).

In order to investigate if transmission of onchocerciasis is ongoing in the West Region of Cameroon, we carried out PCR analysis to assess *Onchocerca* parasites in black flies. Individual black flies were collected in seven communities located in different health districts in the region. The WHO 2016 guidelines for verification of onchocerciasis elimination recommend that a minimum of 6000 black flies must be collected from a transmission zone [13]. Accordingly, in each surveyed community, 2000 black flies were collected giving a total of 14,000 black flies in the transmission zone that we studied. The black flies were collected before ivermectin was distributed in each community by the West Onchocerciasis Control Program. When the required total of 2000 black flies were reached, the collection of flies in the community was stopped. On average, the effective period of fly-catching in each community was five days. The black flies were grouped into pools of 200, separated into heads and bodies giving a total of 10 head pools of 200 heads and 10 body pools of 200 bodies per community. DNA was extracted from each pool and tested for the presence of *Onchocerca* spp. specific DNA using an O-150 PCR assay. End-point agarose electropherogram PCR of positive samples showed three sharp bands (Figure 1) characteristic of the presence of *Onchocerca* spp. DNA in the pools of black flies analyzed [14]. Amplification via in silico PCR (http://insilico.ehu.eus/), predicted that each band has a length of 147 bp with the first, second, and third bands at positions 1, 298, and 595, respectively, of the *Onchocerca* spp. repetitive sequence DNA (GenBank: J04659.1). Following the processing and analysis of all the collected black fly samples, we found that the percentage of infective pools was 70% in Makouopsap, and 0% in all the other communities while the percentage of infected pools was 80%, 30%, and 20% in Makouopsap, Bankambe, and Bayon, respectively, and 0% in Lemgo, Tsesse, Ndionzou, and Kouffen (see Figure 2).

### 2.2. Frequency of Infective and Infected Black Flies

The frequency of the infective and infected black flies—taken from pools of black fly heads and bodies, respectively—were determined using the Poolscreen algorithm. Our findings revealed that the frequency of infective black flies was 0.6% (95% CI 0.00216–0.01347) in Makouopsap and 0.0% (95% CI 0–0.00096) in the other communities. On the other hand, the frequency of infected black flies was 0.8% (95% CI 0.00303–0.1809) in Makouopsap, 0.2% (95% CI 0.00034–0.00521) in Bankambe, 0.1% (95% CI 0.00131–0.00393) in Bayon, and 0.0% (95% CI 0–0.00096) in Lemgo, Tsesse, Ndionzou, and Kouffen (see Table 1).

## 3. Discussion

This study was motivated by the fundamental lack of information as to whether or not mass drug administration (MDA) should be stopped in previously onchocerciasis-endemic communities in the West Region of Cameroon that have been under MDA with ivermectin for the past two decades. To carry out an evaluation of the impact of continuous chemotherapy on the presence of *Onchocerca* spp. detected in black flies, seven communities were chosen based on recent reports [12]. Our findings provide entomological evidence for continuous transmission of *Onchocerca* spp. in Makouopsap and suggests the need to review current CDTI strategies and the need for *O. volvulus* specific entomological assessments to inform the national program.

The frequency of infective and infected black flies was 0.0% (95% CI 0–0.00096) in Lemgo, Tsesse, Ndionzou, and Kouffen. As per the WHO guidelines, in order to confirm interruption of onchocerciasis transmission—for a total of 2000 black flies—the upper bound of the 95% confidence interval of the prevalence of infective black flies should be less than 0.05%, which is equivalent to one infected black fly per 2000 black flies. Moreover, a minimum of 6000 black flies should be collected from a transmission zone and all should be free of infective larvae to ensure that the upper bound of the 95% confidence interval is met [13]. Accordingly, in the present study, a total of 2000 black flies were examined in each community making a total of 14,000 flies from the transmission zone. The results therefore indicate that there is no ongoing transmission of the disease in Lemgo, Tsesse, Ndionzou, and Kouffen and that *Onchocerca* parasites are absent in humans as well as in the cattle population in these communities. These findings are in accordance with the report of Kamga et al. [12], which portrayed a microfilaridermia prevalence of 0% in humans in these communities. Notwithstanding, the entomological evaluation of all neighboring communities is needed before discontinuing annual MDA in these communities to avoid recrudescence of the disease as was the case in Burkina Faso [15]. In this perspective, the use of the Ov-16 serology test to detect exposure to the *O. volvulus* parasite in children below 10 years is highly recommended [16].

There were no infective black flies in Bankambe and Bayon while the percentage of infected black fly pools was 30% and 20%, respectively. Moreover, the frequency of infective black flies was 0.0% (95% CI 0–0.0096) in both communities while the frequency of infected black flies was 0.2% (95% CI 0.00034–0.00521) in Bankambe and 0.1% (95% CI 0.00131–0.00393) in Bayon. These results indicate that the transmission of onchocerciasis has been interrupted in these communities considering that the presence of *Onchocerca* parasites in black fly bodies does not necessarily imply current transmission, which usually requires the presence of infective L3 stage larvae in the head of the fly [13]. The body of black flies, which may contain *Onchocerca* spp. DNA from microfilaria or L2 stages, is used to evaluate the presence of the parasites in humans and cattle [5,13]. Therefore, the frequency of 0.2% and 0.1% of infected black flies—respectively obtained in Bankambe and Bayon—indicates that the parasite is present in the human and/or cattle population. This is in line with the findings of Kamga et al. [12], that reported a microfilaridermia prevalence of 2.6% and 2.3% in humans in Bankambe and Bayon, respectively. Thus, these findings suggest that ivermectin distribution should continue.

In Makouopsap, the percentage of infective and infected black fly pools were 70% and 80%, respectively, with a frequency of 0.6% (95% CI 0.00216–0.01347) and 0.8% (95% CI 0.00303–0.1809), respectively. These results indicate that transmission of onchocerciasis is ongoing in Makouopsap, and this is supported by the high prevalence of nodules and microfilaria reported by others [12,17]. The high frequency of onchocerciasis in Makouopsap may be due to suboptimal responses to ivermectin as was observed in some onchocerciasis endemic areas in the South West Region of Cameroon and Ghana [8,18]. Another possible explanation for the high frequency of the disease could be related to reinfection across the neighboring Center Region, where there are reports of considerable onchocerciasis transmission [19,20]. Therefore, a collaboration between the regions to harmonize intervention strategy is needed in order to reach the goal of eliminating onchocerciasis transmission. Additionally, new strategic options and alternative treatment strategies should be implemented to accelerate the elimination of onchocerciasis transmission in Makouopsap. A probable elimination strategy could be the use of bi-annual MDA of ivermectin as was suggested by APOC [21]. This strategy successfully interrupted the transmission of *O. volvulus* in the Abu Hamed focus in Sudan [22]. Taken together, there is a need for the development of new treatment strategies to combat this disease in order to achieve the goal of elimination [23,24].

The authors acknowledge the limitation of not using *O. volvulus*-specific DNA probes to detect *O. volvulus*. Indeed, it has been mentioned that the use of *O. volvulus*-specific DNA probes guarantees absolute specificity and allows for processing large numbers of black flies; thereby, increasing the reliability of the results [25]. The entomological evidence reported herein reveals that there is on-going transmission of onchocerciasis in Makouopsap. However, since *O. volvulus* specific DNA probes were not employed, there is a possibility that some of the parasites found in the black flies could be *O. ochengi*, as was reported in the North Region of Cameroon [26]. Notwithstanding, the high proportion of individuals infected with *O. volvulus* as reported by Kamga et al. [12], do suggest that the majority of the black flies may be infected with *O. volvulus*.

Another limitation of this study is that it did not cover all the onchocerciasis endemic communities in the West Region of Cameroon. Our near-future goal is to use the *O*. *volvulus*-specific DNA probes to detect *O*. *volvulus* in samples from the other endemic communities of the transmission zone. Although this study has some limitations, it addresses a gap in the literature. Thus far, the scant data on the West Region of Cameroon CDTI program have not addressed entomological indices. Our attempt to detect *Onchocerca* spp. DNA in black flies offers insight into the entomological profile of onchocerciasis transmission in the West Region of Cameroon and makes our study complementary to previous parasitological studies that were carried out in the region.

## 4. Conclusions

The data presented demonstrate that the CDTI strategy for the treatment of onchocerciasis in Cameroon has made progress towards the elimination of transmission of this disease in Lemgo, Tsesse, Ndionzou, Kouffen, Bankambe, and Bayon. Transmission of onchocerciasis is ongoing in Makouopsap, and to reach the goal of interrupting transmission completely, it will be necessary for the program to adopt new strategic options and alternative treatment strategies.

## 5. Materials and Methods

### 5.1. Ethical Considerations and Participant Enlistment

The study was approved by the Cameroon Bioethics Initiative (CAMBIN) Ethics Review and Consultancy Committee (ERCC) (N°CBI/443/ERCC/CAMBIN). Administrative authorization was obtained from the Cameroon Ministry of Public Health (N°631–1315). Written informed consent forms were distributed and well explained to all black fly collectors. All the black fly collectors, who were indigenes of the respective studied communities, voluntarily signed and returned the consent form before participation in the study. Ivermectin was administered to all black fly collectors one week prior to the start of collection.

### 5.2. Selection of Study Sites and Black Fly Collection

**Figure 3:**
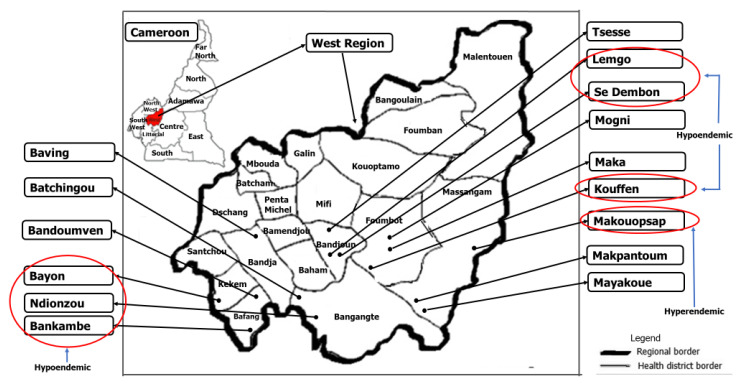
Map of the West Region of Cameroon showing the surveyed communities. The communities were selected based on the prevalence of microfilaria in humans that we reported earlier [12].

This study was conducted in seven communities in the West Region of Cameroon located in the same transmission zone. As per the WHO guidelines, a transmission zone is a geographical area where transmission of *O. volvulus* occurs by locally breeding vectors and which can be regarded as a natural ecological and epidemiological unit for interventions [13]. The black flies were identified by an entomologist based on the morphological characters used for the identification of black fly species: the color of the scutellum, wing tufts and arculus of the antennae, the procoxa, and the ninth abdominal tergit [27]. The black fly species in the area was the *Simulium squamosum,* which belongs to the forest cytospecies. Entomological collections were conducted during the peak transmission period of August through November 2019 in Makouopsap, Bankambe, Bayon, Lemgo, Tsesse, Ndionzou, and Kouffen (see Figure 3)—located in 6 out of the 20 health districts of the West Region of Cameroon (5°30′0″ N, 10°30′0″ E)—based on recent reports on parasitological findings in humans after 21 years of CDTI in these communities [12]. In principle, all the 15 communities were planned for testing since they all shared the same vector. However, we decided to start with six communities that were reported to be near elimination and one which is hyperendemic as a control (see Table 2). The communities were considered first line because they are found near the breading site of the vector. Treatment of onchocerciasis in these communities is done yearly in August [28], but with delays, treatment can span through the whole peak transmission period. The standard human landing capture method [29] was used to obtain 2000 black flies during daytime in each surveyed community. Briefly, the black fly catchers were separated into groups composed of one human bait and two collectors. The human baits wore black long thick socks that cover their entire legs and sat on higher stools while the collectors sat on lower stools and wait for flies to land on the lower limbs of the baits. Once a black fly lands on a bait, the collector covers it with a tube and captures it as it tries to escape. The collector covers the tube with the finger and quickly puts the cork mesh and then the cork on the tube to trap the black fly. All black flies showing evidence of a recent blood meal were discarded. Black flies caught were drowned for 2 min using a drowning solution (250 μL Kodak PhotoFlo-200 and 500 mL 1× PBS) and transferred to a cleaning solution (500 μL Tween-80, 500 μL 5% Clorox, 10 mL 100× PSF, and 489 mL 1× PBS). The black flies were then stored in 100% iso-propanol in pools of 200 at room temperature [30] until transported to the Molecular and Cell Biology laboratory (MCBL) of the University of Buea, Cameroon. The preserved black fly pools were then placed in liquid nitrogen and subjected to vigorous agitation to separate the heads from the bodies. The heads were collected by sieving through 25 mesh size sieves. The mesh has been reported to be effective in separating black fly bodies and heads without cross contamination [30,31,32]. Additionally, a clean separation was verified using a dissection microscope (Olympus, Tokyo, Japan) and the heads and bodies were stored separately in pools of 200 each at −80 °C.

### 5.3. DNA Extraction

The pool of samples was rinsed three times in 95% ethanol. Thereafter, separated heads and bodies were frozen in liquid nitrogen and macerated with pestles. DNA was extracted from each pool using Maxwell S3000 robot (Promega Co., Madison, WI, USA) and the Maxwell^®^ RSC Pure food, GMO, and Authentication kit (Promega Co., Madison, WI, USA) according to manufacturer’s instruction. Briefly, 600 µL of CTAB Buffer, 2 µL of RNase A and 30 µL of Proteinase K were added to a 1.5 mL microcentrifuge tube containing 200 mg of sample. The samples were incubated at 60 °C for 30 min and centrifuged at room temperature for 10 min at 13,000 RPM. Thereafter, 300 µL of clear lysate sample was transferred to the reagent cartridge which was placed in the Maxwell^®^ 16 for automated DNA purification. This technique has been used to extract DNA from samples of diverse origin including black flies [31,33,34,35]. Moreover, the sensitivity and relative effectiveness of the method was evaluated in comparison with the phenol chloroform method—gold standard of DNA extraction for entomological surveillance in Latin America—for the detection of *O. volvulus* in pools of black flies, with results indicating that the automated method is as sensitive as phenol chloroform method, nontoxic, and rapid [31].

### 5.4. Amplification by PCR

An O-150 PCR (Poolscreen) approach was used. This technique is an up-to-date technique for *Onchocerca* spp. detection in black flies. It was recently used for the entomological assessment of transmission following recrudescence of onchocerciasis in the Comoé Valley, Burkina Faso [15]. Moreover, the technique has also been employed for the detection of *Onchocerca* spp. by several authors [5,22,32,36,37,38]. A set of PCR primers comprising the forward primer, (5′-ATCAATTTTGCAAAATGCG-3′) and the reverse primer, (5′-AATAACTGATGACCTATGACC-3′) that flank the region of interest in *Onchocerca* spp. specific gene was used for PCR amplification [14]. Amplification via in silico PCR (http://insilico.ehu.eus/), predicted that there can be a maximum of 4 PCR amplicon, each of length 147 bp at positions 1, 298, 595, and 1189 of the *Onchocerca* spp. repetitive sequence DNA (GenBank: J04659.1). The PCR was performed using ReadyMix^TM^ Taq PCR Mastermix (Sigma, Neustadt an der Weinstraße, Germany) in a total volume of 15 μL. The standard PCR conditions were 95 °C for 3 min followed by 35 cycles at 95 °C for 1 min, 45 °C for 30 s, and 72 °C for 1 min, with a final extension at 72 °C for 10 min. Samples that generated a PCR signal on the electropherogram were considered positive. Only samples that were positive after a second independent PCR were reported as confirmed positive. This method was reported to be specific for detecting *Onchocerca* spp. in pools of black flies and additional purification steps of genomic DNA did not influence the PCR results obtained [14].

### 5.5. Data Analysis

A maximum likelihood approach was applied to estimate the frequency of infection—in the vector population—and the confidence intervals using the algorithms contained in the Poolscreen software [39]. Two sets of analyses were performed to calculate the infection rates in the vector population. These included the frequency of infective black flies—calculated from pools of heads—representing black flies harboring infective L3 larval stage and the frequency of infected black flies, calculated from pools of bodies, representing black flies harboring non-infectious L1 and L2 stages [40]. Chi square test was used to compare the proportions of black flies between the communities. On the other hand, the risk of infective or infected status of black flies was assessed by binary logistic regression using SPSS v26. A *p*-value < 0.05 was considered statistically significant.

## Figures and Tables

**Table 1:** Frequency of infective and infected black flies determined by the Poolscreen algorithm with 95% Confidence Interval (CI) in pools of 200 black flies.

Community	Infective Pool		Infected Pool	
	Frequency	95% Confidence Interval ^a^	Frequency	95% Confidence Interval ^a^
Bankambe	0.0	0–0.00096	0.2	0.00034–0.00521
Lemgo	0.0	0–0.00096	0.0	0–0.00096
Tsesse	0.0	0–0.00096	0.0	0–0.00096
Ndionzou	0.0	0–0.00096	0.0	0–0.00096
Kouffen	0.0	0–0.00096	0.0	0–0.00096
Bayon	0.0	0–0.00096	0.1	0.00131–0.00393
Makouopsap	0.6	0.00216–0.01347	0.8	0.00303–0.1809

^a^ The flanking values represent the 95% confidence interval of the frequency estimate.

**Table 2:** Prevalence of *O. volvulus* microfilaria in selected communities in 2015.

Health District	Community	*n*	Mf + (*n*)	Microfilaria Prevalence (%)
Bafang	Bankambe	203	9	2.6
Bandjoun	Lemgo	75	0	0.0
	Tsesse	123	0	0.0
Bangangté	Ndionzou	141	0	0.0
Foumbot	Kouffen	107	0	0.0
Kékem	Bayon	231	6	2.3
Massangam	Makouopsap	123	53	41.6

Microfilaria prevalence in the surveyed communities after 21 years of CDTI [12]. *n* = number examined, Mf + (*n*) = number with microfilaria positive skin snip.
